# Graphene Oxide Bulk-Modified Screen-Printed Electrodes Provide Beneficial Electroanalytical Sensing Capabilities

**DOI:** 10.3390/bios10030027

**Published:** 2020-03-19

**Authors:** Samuel J. Rowley-Neale, Dale A. C. Brownson, Graham Smith, Craig E. Banks

**Affiliations:** 1 Faculty of Science and Engineering, Manchester Metropolitan University, Chester Street, Manchester M1 5GD, UK; S.Rowley-Neale@mmu.ac.uk (S.J.R.-N.); d.brownson@mmu.ac.uk (D.A.C.B.); 2 Department of Natural Sciences, Faculty of Science and Engineering, University of Chester, Thornton Science Park, Pool Lane, Ince, Chester CH2 4NU, UK; graham.smith@chester.ac.uk

**Keywords:** graphene oxide, electroanalytical sensing, dopamine, uric acid, screen-printed electrodes

## Abstract

We demonstrate a facile methodology for the mass production of graphene oxide (GO) bulk-modified screen-printed electrodes (GO-SPEs) that are economical, highly reproducible and provide analytically useful outputs. Through fabricating GO-SPEs with varying percentage mass incorporations (2.5%, 5%, 7.5% and 10%) of GO, an electrocatalytic effect towards the chosen electroanalytical probes is observed, which increases with greater GO incorporated compared to bare/graphite SPEs. The optimum mass ratio of 10% GO to 90% carbon ink produces an electroanalytical signal towards dopamine (DA) and uric acid (UA) which is ca. ×10 greater in magnitude than that achievable at a bare/unmodified graphite SPE. Furthermore, 10% GO-SPEs exhibit a competitively low limit of detection (3σ) towards DA at ca. 81 nM, which is superior to that of a bare/unmodified graphite SPE at ca. 780 nM. The improved analytical response is attributed to the large number of oxygenated species inhabiting the edge and defect sites of the GO nanosheets, which are able to exhibit electrocatalytic responses towards inner-sphere electrochemical analytes. Our reported methodology is simple, scalable, and cost effective for the fabrication of GO-SPEs that display highly competitive LODs and are of significant interest for use in commercial and medicinal applications.

## 1. Introduction

Graphene oxide (GO), a two-dimensional oxygenated carbon nanosheet, previously considered by many researchers as solely a precursor for the synthesis of graphene, possesses a number of unique chemical properties that make it a beneficial material in its own right [1,2,3]. Whilst researchers have found niche applications for GO in an array of technologies, such as hydrogen storage [4], supercapacitors [5], and biosensors [6], GO is often overlooked due to its limited application in electrically active devices/materials. This is a result of its reported high electrical resistance that stems from carboxyl, hydroxyl, and epoxy groups located on the periphery of the GO sheet [7]. It is however, these hydrophilic oxygenated functional groups which assist in biorecognition during biosensing by promoting favourable interactions with specific analytes [1,8,9,10], allowing GO to be used as the underlying electrode material for a biosensor towards a number of biological/organic molecules, such as DNA [11,12] and peptides [13]. In many cases where GO is utilised for sensing applications, it is as a component/supporting framework within a more complex catalyst [14,15]. GO’s ability to act singularly as a (bio)sensor has yet to be observed within the literature. A study by Brownson et al. [16] demonstrated that GO, when immobilised upon the surface of graphitic electrodes, exhibited intriguing electrochemical responses, with the redox probes studied giving rise to electrochemical responses dependent upon the C/O content [17]. This suggests that GO could be beneficially utilised as an electrochemical platform where oxygenated electrocatalytic reactions are involved. 

In this paper, we take this prior work one-step further [17] by fabricating GO bulk-modified SPEs and explore their performance towards a range of electroanalytically interesting analytes, namely dopamine (DA) and uric acid (UA). The preferred method of detection is via electrochemical techniques, as they offer rapid, portable and low cost analysis. It is evident that the literature focuses (See Table 1) on graphene rather than GO as an electrochemical sensing platform, where the chosen nanomaterial is drop casted upon a supporting carbon electrode, allowing it to be electrochemically wired. The use of drop casting as a method to modify a supporting electrode has several drawbacks, such as the supporting electrode has to be prepared for each measurement, which can be time consuming, and the drop-casting process results in an uncontrollable distribution of the nanomaterial upon the electrode’s surface, that in turn results in poor reproducibility [18,19]. In order to overcome these issues, screen-printed electrodes (SPEs) have proven to be mass-producible electrochemical sensing platforms that offer versatility in electrode design and repeatability in the signal output [20]. The screen-printing technique can produce a vast number of SPEs that exhibit uniform heterogeneous electron transfer kinetics, thereby enabling separate electrodes to be used for independent measurements and give consistent/reliable responses. SPEs can also be readily adapted with respect to the composition of the ink utilised in their production, allowing for the incorporation of materials that alter the electrocatalytic behaviour displayed by the SPE [18].

In order to explore this principle, this paper reports the bulk modification of SPEs, with varying percentage mass incorporations of GO and electrochemically exploring the capabilities of GO bulk-modified screen-printed electrodes (GO-SPEs), in comparison to bare/unmodified SPEs, as potential electroanalytical sensing platforms towards DA and UA (separately) for the first time.

## 2. Experimental Section

All chemicals used were of analytical grade and were used as received from Sigma-Aldrich without any further purification. All solutions were prepared with deionised water of resistivity no less than 18.2 MΩ cm^–1^ and were vigorously degassed prior to electrochemical measurements with high purity, oxygen free nitrogen. The GO powder utilised was commercially purchased from Graphene Supermarket [26].

Electrochemical measurements were performed using an Ivium CompactstatTM (Eindhoven, The Netherlands) potentiostat. Measurements were carried out using a typical three-electrode system, with a Pt wire counter electrode and a saturated calomel electrode (SCE) reference. The working electrodes were screen-printed graphite electrodes (SPEs), which have a 3.1 mm diameter working electrode. The SPEs were fabricated in house, the methodology of which is outlined in the electronic supporting information (ESI). Following production of the standard SPE, modification/production of the GO variation was achieved as follows: the GO powder was incorporated into the bulk graphitic ink on the basis of the weight percentage of MP to MI, where MP is the mass of particulate (in this case the GO) and MI is the mass of the ink formulation used in the printing process, i.e., % = (MP/MI) × 100. The weight percentage of MP to MI varied from 2.5%, 5%, 7.5% to 10%, resulting in 4 separate GO bespoke inks that are then screen printed upon the working area of bare SPEs; see the ESI for further details. Note, the maximum amount of GO that can be incorporated into the graphitic ink was found to correspond to 10% with any further percentage incorporation resulting in an increase in the resultant ink viscosity to where it is not screen printable via the technique used within this manuscript.

Physicochemical characterisation was performed utilising Raman spectroscopy, transmission electron microscopy (TEM), X-ray diffraction (XRD) and X-ray photoelectron spectroscopy (XPS). Details of the instrumentation utilised are reported in the ESI.

## 3. Results and Discussion

**Figure 1:**
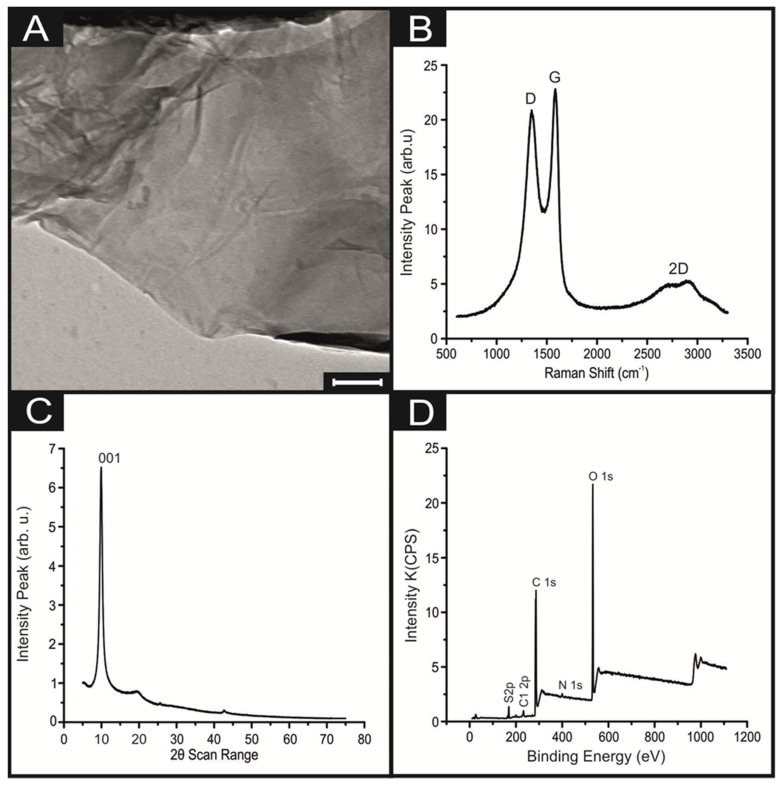
Characterisation of the commercially sourced GO; (**A**) image of the GO nanosheet (Scale bar: 100 nm), (**B**) Raman spectra of GO deposited onto a silicon wafer between 100 and 3400 cm, (**C**) X-ray diffraction (XRD) spectra between 5 and 75 2*θ*, and (**D**) high-resolution XPS survey spectra.

Initially, it was essential to perform a full physicochemical characterisation of the commercially purchased GO powder in order to ascertain its quality/properties prior to being incorporated into the SPEs (as reported in the experimental section). Raman spectroscopy, SEM, TEM, XPS and XRD analysis were all conducted. Figure 1A displays a TEM of the GO nano-platelets indicating that they exhibit a particle size (lateral width) of between 300 and 600 nm, which strongly agrees with the size stated by the commercial manufacturer, of ca. 500 nm [26]. 

Next, Raman spectroscopy was utilised to confirm the presence of GO by structural characterisation. The obtained spectra can be viewed in Figure 1B and displays the D and G vibrational band peaks at ca. 1350 and 1590 cm^–1^, respectively; which are typically characteristic of GO [27,28]. Additionally, the composition of the GO sample is confirmed via XRD in Figure 1C, in which a characteristic ‘sharp’ peak is evident at 2θ = 11.5°, corresponding to the (001) diffraction peak of disordered GO [29]. Lastly, XPS analysis was performed to determine the GO’s elemental composition, with Figure 1D showing the gathered survey spectra and Figure S1 displaying the individual spectra for the C and O regions. The GO was observed to contain 66.8% carbon and 28.6% oxygen, with trace amounts of nitrogen, sulphur and chlorine, which are likely mere contaminants. The combination of surface and physicochemical analysis presented above and expanded upon within the ESI confirm that the commercially sourced GO herein utilised is of high quality/purity. 

The GO-SPEs (the design and fabrication of which are outlined within the ESI) were electrochemically evaluated using the near ideal ‘outer-sphere’ redox probe 1 mM [Ru(NH_3_)_6_]^3+/2+^ in 0.1 M KCl [30]. SEM was utilised to image the surface of a bare SPE and a 10% GO-SPE. However, the obtained images were indistinguishable due to the GO nanosheets having a very similar appearance to graphitic nanoplatelets found within the SPE bulk ink (see Figure S2). Whilst the bare/unmodified SPEs and the GO-SPEs were visually indistinguishable at the microscale, the incorporation of the GO into the SPEs bulk ink significantly altered their electrochemical performance, as described below. Utilising a 10% GO-SPE as a representative example, the observed voltammetric profiles are presented in Figure S3. Note that the electrochemical reduction peak current increased from 3.6 to 32 µA on the bare SPE compared to the 10% GO-SPE, respectively. Note, however, that the 10% GO-SPE displayed a smaller oxidation peak than the bare SPE. This alteration in the obtained cyclic voltammetric (CV) response is characteristic of an EC’ type reaction as described previously by Brownson et al. [16], who explored the electrochemistry of GO towards select redox probes by drop casting it onto an edge plane pyrolytic graphite (EPPG) support electrode. Such a response suggests that, as the amount of GO incorporation into the GO-SPEs is increased, so too is the proportion of oxygenated species present, resulting in a larger amount of oxygenated species available to catalyse the chemical reaction. Note that the electrochemical response of “graphene” towards [Ru(NH_3_)_6_]^3+/2+^ does not display the catalytic behaviour herein observed at GO [31]. This inference could allow for an electrochemical test to differentiate the presence of “true” graphene and GO, as they have unique CV signal responses. The proposition that it is the C-O groups that produce such a response is as pointed out by Brownson et al. [17], who observed similar electrochemical signatures [16], making GO a much more promising electrocatalyst for sensing applications than graphene—especially when the amount and coverage of GO is highly controlled, as is the case with the GO-SPEs produced herein. 

Next, the electroanalytical efficacy of the GO-SPEs was explored towards the sensing of dopamine (DA). DA is a neurotransmitter essential for bodily functions, such as memory and emotional regulation [32,33], where the detection of DA within body fluids is widely studied, as its concentration within bodily systems is linked to numerous neurological disorders [16].

**Figure 2:**
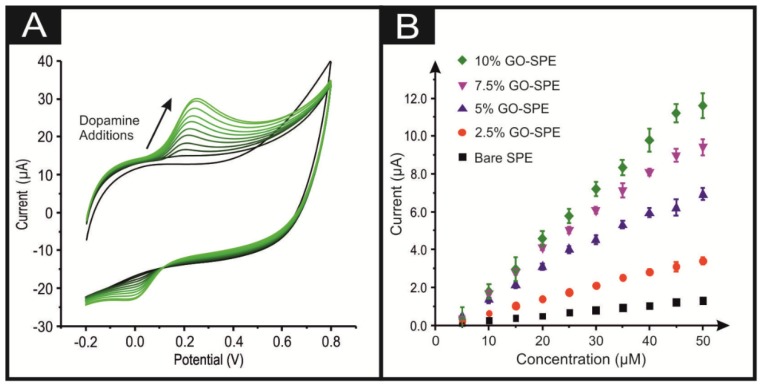
(**A**) Typical cyclic voltammetric response obtained utilising 10% GO-SPEs by sequentially adding aliquots of DA into pH 7.4 PBS, from 5 to 50 µM. (**B**) Calibration plot of the anodic peak current associated with the electroanalytical oxidation of DA over the concentration range for a bare SPE (black square), a 2.5% GO-SPE (orange circle), a 5% GO-SPE (blue triangle), a 7.5% GO-SPE (purple inverted triangle), and a 10% GO-SPE (green star). Error bars are on the data points and represent the average standard deviation (N = 3). Scan rate utilised: 100 mVs^–1^ (vs. SCE).

**Figure 3:**
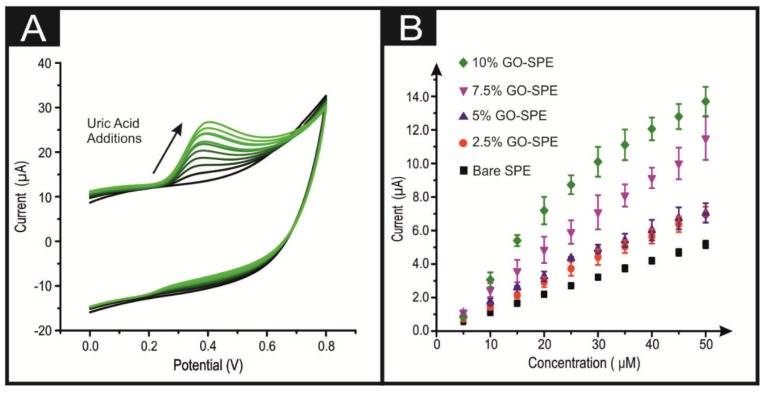
(**A**) Typical cyclic voltammetric response obtained utilising 10% GO-SPEs by sequentially adding aliquots of UA to pH 7.4 PBS, from 20 to 200 µM. (**B**) Calibration plot of the anodic peak current associated with the oxidation of UA over the concentration range for a bare SPE (black square), a 2.5% GO-SPE (orange circle), a 5% GO-SPE (blue triangle), a 7.5% GO-SPE (purple inverted triangle), and a 10% GO-SPE (green star). Error bars are on the data points and represent the average standard deviation (N = 3). Scan rate utilised: 100 mVs^–1^ (vs. SCE).

Additions of DA were made into to a phosphate buffer (pH 7) solution, incrementing the DA concentration from 5 to 50 µM. The obtained CVs and calibration plots are presented within Figure 2. Using the 10% GO-SPEs as a representative example of all the GO-SPEs, Figure 2A shows that the oxidation peak current at a 5 µM DA concentration was 1.21 µA, which subsequently increased to 15.24 µA by 50 µM. There was a corresponding anodic shift in the onset potential from + 0.212 to + 0.316 V (all values are deduced from an average of N = 3). Of note is the large capacitive effect observed when GO is incorporated into the bulk of the SPEs (see Figure 2 and Figure 3). This is to be expected, as previous literature has noted GO’s capacitive nature [34]. The bare/unmodified SPEs do not display this capacitive effect (see Figures S4 and S5). It is clearly observable from Figure 2B that in agreement with the 10% GO-SPE, all the GO-SPEs display a greater anodic peak current than the bare SPE (see Figure S4). This can be associated with the oxygenated species present on GO facilitating the oxygenated electrocatalytic reactions. This is further supported by the observation that the greater percentage incorporation of GO into the GO-SPE the larger the observed anodic peak current (see Figure 2B). However, as the percentage of GO within the electrode increases from 0 to 10%, the activation potential for DA oxidation increases. A similar trend was observed when UA was utilised in the exact manner as above rather than DA (see Figure 3 and Figure S5), with a 10% GO-SPE displaying a ca. ×10 increase in the achievable peak current density when compared to a bare SPE. For a full description, see the ESI.

In terms of the analytical utility of the GO-SPE towards DA and UA sensing, there is a clear correlation between the percentage mass incorporation of GO and the electrode’s limit of detection. Of note is the appearance of two linear ranges within a number of the trend lines for the separate electrodes in Figure 2 and Figure 3. In these cases, the initial linear range was utilised as the slope for LOD calculations. As shown in Table 1, a bare/unmodified SPE displays an analytical useful limit of detection (LOD, based on 3σ) for DA and UA at 0.78 and 2.3 µM respectively. The 10% GO-SPE exhibited the lowest limit of detection of 81 nM and 0.61 µM for DA and UA respectively. The LOD values for the GO-SPE are highly competitive to those found within the current literature. They are also within the medically relevant range, given that the baseline concentration of DA within the striatum is ca. 10–20 nM, with unusual activity (i.e., burst firing) associated with neurological disorders exhibited by high DA concentrations in the hundreds of µM range [35]. The above observations suggest that the synergy between GO and the SPE offers huge beneficial electrocatalytic responses towards DA. 

The intra-repeatability of the GO-SPEs was tested (N = 3). The percentage relative standard deviation (%RSD) for the observed peak current observed at the bare/unmodified SPE, 2.5%, 5%, 7.5%, and 10% GO-SPEs is shown via error bars in Figure 2B and Figure 3B. With respect to the observed oxidation peak current, there is clearly a trend of increasing %RSD corresponding to an increase in the percentage of GO within the GO-SPEs. We postulate that this is due to a greater percentage of GO present, leading to a larger number of variations within the orientation of the modified GO structure, whereby there will be a greater chance for a different proportional of the GO oxygenated species to be present on the electrodes surface. The %RSDs at 50 µM for the bare/unmodified SPE, 2.5%, 5%, 7.5%, and 10% GO-SPEs are 1.7, 2.2, 3.4, 5.1 and 5.8 percent, respectively. These low %RSD values for the anodic oxidation peak attest to the high/favourable reproducibility of the screen-printing technique utilised herein to produce the GO-SPEs.

## 4. Conclusions

We have designed, fabricated and evaluated GO bulk-modified SPEs, which demonstrate electrocatalytic capabilities towards the sensing of DA and UA. The application of GO in this manner takes advantage of the oxygenated surface species inhabiting the edge and defect sites of the GO nanosheets to create a cheap, mass producible and tailorable sensing platform for applications requiring oxygenated electrocatalysis. Through increasing the amount of GO present (to a maximum of 10%), we observe a correlation between the number of oxygenated species and the magnitude of DA and UA electroanalytical signals. 

## Figures and Tables

**Table 1:** Comparison of current literature reporting the use of graphene and related electrocatalytic materials explored towards the electroanalytical sensing of dopamine (DA) and uric acid (UA).

Electrocatalyst	Electrode Material	Deposition Technique	Dopamine LOD (M)	Uric Acid LOD (M)	Electrochemical Method	Reference
GO-MWCNT/MnO_2_AuNP	GC	Drop Cast	1.7 × 10^–7^	–	CV	[14]
pCu_2_O NS-rGO	GC	Drop Cast	1.5 × 10^–8^	1.1 × 10^–7^	DPV	[21]
G-SnO_2_	GC	Drop Cast	1.0 × 10^–6^	–	DPV	[22]
DA-ERG/PMB	GC	Drop Cast	1.0 × 10^–7^	–	DPV	[23]
GSCR-MIPs	GC	Drop Cast	1.0 × 10^–7^	–	LSV	[24]
NG	GC	Drop Cast	2.5 × 10^–7^	4.5 × 10^–8^	DPV	[25]
Bare/unmodified	SPE	Screen Printed	7.8 × 10^–7^	2.3 × 10^–6^	CV	This Work
2.5% GO-ink	SPE	Screen Printed	2.9 × 10^–7^	1.6 × 10^–6^	CV	This Work
5% GO-ink	SPE	Screen Printed	1.3 × 10^–7^	1.0 × 10^–6^	CV	This Work
7.5% GO-ink	SPE	Screen Printed	1.0 × 10^–7^	9.6 × 10^–7^	CV	This Work
10% GO-ink	SPE	Screen Printed	8.1 × 10^–8^	6.1 × 10^–7^	CV	This Work

GC, glassy carbon; GO-MWCNT/MnO_2_AuNP, graphene oxide multi-walled carbon nanotubes with manganese dioxide, poly(diallyldimethylammonium chloride) and gold nanoparticles; –, value unknown or not applicable; CV, cycling voltammetry; pCu_2_O NS-rGO, porous cuprous oxide nanospheres on reduced graphene oxide; DPV, differential pulse voltammetry; G-SnO_2_, graphene-tin oxide; DA-ERG/PMB, dopamine-grafted reduced graphene oxide/poly(methylene blue); GSCR-MIPs, graphene sheets/Congo red molecular imprinted polymers; LSV, linear sweep voltammetry; NG, nitrogen doped graphene; SPE, screen-printed electrode.

## Supplementary Materials

The following are available online at https://www.mdpi.com/2079-6374/10/3/27/s1. Includes the following sections: Electrode production, Experimental details on physicochemical characterization, Scan rate study, Dopamine electrochemistry, and Uric acid electrochemistry. Supporting Information figures: Figure S1. High-resolution XPS spectra of C and O regions of the GO utilised herein (A and B respectively). Figure S2. SEM images of the graphite and GO electrode surfaces in the supercapacitor device show little variation in the surface morphology of the surfaces with variation in GO content. Given this, it is apparent that the dominating influence of the morphology of the electrodes is in fact the carbon ink. This indicates that the improvement in the performance is a result of physicochemical properties of the graphene oxide, and not a result of any morphological differences induced by the addition of the GO. Figure S3. Typical cyclic voltammetric response of a bare SPE and a 10% GO-SPE recorded 1 mM [Ru(NH_3_)_6_]^3+/2+^ in 0.1 M KCl solution. Scan rate utilised: 5 mVs^–1^ (vs. SCE). Figure S4. Typical cyclic voltammetric response obtained utilising a Bare/unmodified SPE by sequentially adding aliquots of 0.5 mM DA to pH 7.4 PBS, additions from 5 to 50 µM. Figure S5. Typical cyclic voltammetric response obtained utilising a bare/unmodified SPE by sequentially adding aliquots of 2 mM UA to pH 7.4 PBS, altering the bulk solution from 20 to 200 µM.
