# Supplementary material for: Graphene Oxide Bulk-Modified Screen-Printed Electrodes Provide Beneficial Electroanalytical Sensing Capabilities
Source: Biosensors (Basel). 2020 Mar 19;10(3):27. doi: 10.3390/bios10030027 (PMC7146522; doi:10.3390/bios10030027)
Supplement: Supplementary file 1 [file biosensors-10-00027-s001.pdf]

Electronic Supporting Information (ESI) for the following publication:

Graphene Oxide Bulk-Modified Screen-Printed Electrodes Provide Beneficial Electroanalytical Sensing Capabilities

Samuel J. Rowley-Neale [1], Dale A. C. Brownson [1], Graham Smith [2] and Craig E. Banks [1,*]

[1] Faculty of Science and Engineering, Manchester Metropolitan University, Chester Street, Manchester M1 5GD, UK.

[2] Faculty of Science and Engineering, Department of Natural Sciences, University of Chester, Thornton Science Park, Pool Lane, Ince, Chester CH2 4NU, UK.

* Correspondence: c.banks@mmu.ac.uk; Tel: +(0)−161−2471−196; Fax: +(0)−161−2476−831

1. Electrode Production

The GO incorporated ink formulations described within the main manuscript were printed using the appropriate stencils by a DEK 248 screen-printing machine (DEK, Weymouth, U.K.) [1]. These electrodes have been used extensively in previous studies [2–6]. In their fabrication; first a carbon-graphite ink formulation (product code C2000802P2; Gwent Electronic Materials Ltd., U.K.) was screen-printed onto a polyester (Autostat, 250 μm thickness) flexible film This layer was cured in a fan oven at 60 °C for 30 min. Next, a silver/silver chloride reference electrode was included by screen-printing Ag/AgCl paste (product code C2030812P3; Gwent Electronic Materials Ltd., U.K.) onto the polyester substrates and a second curing step was undertaken where the electrodes were cured at 60 °C for 30 min. Finally, a dielectric paste (product code D2070423D5; Gwent Electronic Materials Ltd., U.K.) was then printed onto the polyester substrate to cover the connections. After a final curing at 60 °C for 30 min the SPEs are ready to be used and were connected *via* an edge connector to ensure a secure electrical connection [2]. The unmodified SPEs have been reported previously and shown to exhibit a heterogeneous electron transfer rate constant, k^o, of *ca*. 10^{-3} cm s^{-1}, as measured using the $[Ru(NH_3)_6]^{3+/2+}$ outer-sphere redox probe [3]. The GO was incorporated into the bulk of the SPEs on the basis of the weight percent of M_P to M_I, where M_P is the mass of particulate (in this case the GO) and M_I is the mass of the ink formulation used in the printing process, *i.e.* % = (M_P / M_I) × 100. The weight percent of M_P to M_I varied from 2.5, 5, 7.5 and 10%, which resulted four separate inks that could subsequently be individually screen-printed on top of the working SPE electrode (see above) and cured as described earlier (60 °C for 30 min). Note that for the purpose of this work, electrochemical experiments were performed using the working electrode of the SPEs only and external reference and counter electrodes were utilised as detailed earlier to allow a direct comparison between all the electrodes utilised and with prior literature.

2. Experimental: Physicochemical Characterisation

Transmission electron microscopy (TEM) images were obtained using a 200 kV primary beam under conventional bright-field conditions. The 2D-$MoSe_2$ sample was dispersed onto a holey-carbon film supported on a 300 mesh Cu TEM grid. Raman Spectroscopy was performed using a 'Renishaw InVia' spectrometer equipped with a confocal microscope (×50 objective) and an argon laser (514.3 nm excitation). Measurements were performed at a very low laser power level (0.8 mW) to avoid any heating effects. X-ray diffraction (XRD) was performed using an "X`pert powder PANalytical" model with a copper source of K_α radiation (of 1.54 Å) and K_β radiation (of 1.39 Å), using a thin sheet of nickel with an absorption edge of 1.49 Å to absorb K_β radiation. A reflection transmission spinner stage (15 rpm) was implemented to hold the commercially sourced GO nano-powder. The range was set between 5 and 80 2θ in correspondence with literature ranges [9]. Additionally, to ensure well

defined peaks an exposure of 50 s per 2θ step was implemented with a size of 0.013°. The x-ray photoelectron spectroscopy (XPS) data was acquired using a bespoke ultra-high vacuum system fitted with a Specs GmbH Focus 500 monochromated Al Kα X-ray source, Specs GmbH Phoibos 150 mm mean radius hemispherical analyser with 9-channeltron detection, and a Specs GmbH FG20 charge neutralising electron gun [4]. Survey spectra were acquired over the binding energy range 1100 – 0 eV using a pass energy of 50 eV and high resolution scans were made over the C 1s and O 1s lines using a pass energy of 20 eV. Under these conditions the full width at half maximum of the Ag 3d$_{5/2}$ reference line is *ca.* 0.7 eV. In each case, the analysis was an area-average over a region approximately 1.4 mm in diameter on the sample surface, using the 7 mm diameter aperture and lens magnification of ×5. The energy scale of the instrument is calibrated according to ISO 15472, and the intensity scale is calibrated using an in-house method traceable to the UK National Physical Laboratory [5]. Data were quantified using Scofield cross sections corrected for the energy dependencies of the electron attenuation lengths and the instrument transmission [6]. Data interpretation was carried out using CasaXPS software v2.3.16 [7].

3. Scan Rate Study

Examining a 10% GO-SPE voltammetric response to 1 mM $[Ru(NH_3)_6]^{3+}$ in 0.1 M KCl at varying scan rates. The voltammetric peak height (I_P) at each scan rate was monitored (υ) with a plot of peak height versus square-root of the scan rate revealing a linear response (I_P (A) = 2.81 x 10^{-4} A/(Vs^{-1})$^{0.5}$; R^2 = 0.87) indicating a diffusional process; furthermore, as is expected for the case of the semi-infinite diffusion model as governed by the Randles–Ševćik equation, analysis of log *Ip* versus log *n* revealed a gradient of 0.4, indicating the absence of thin-layer effects.

4. Dopamine Electrochemistry

Figure S4 depicts the typical CVs obtained when utilising an SPE as a working electrode whilst the concentration of DA (in pH 7.4 phosphate buffer solution (PBS)) is increased from 5 μM to 50 μM. It is clear that the anodic oxidation peak obtained at 5 μM has a peak current of 0.29 μA at *ca.* + 0.198 V. Successive increases in the concentration of DA corresponded to an increase in the peak current as well as a slight anodic shift in the peak potential until at 50 μM they were observed to be 2.74 μA and + 0.257 V respectively.

5. Uric Acid Electrochemistry

We will now explore the GO-SPEs towards UA. Firstly it was essential to bench mark our system by using a bare/unmodified SPE and examining its ability to detect UA. Figure S5 depicts the typical CVs obtained when utilising an SPE as a working electrode whilst the concentration of UA (in pH 7.4 phosphate buffer solution (PBS)) is increased from 20 μM to 200 μM. It is clear that the anodic oxidation peak obtained at 20 μM has a peak current of 0.53 μA at *ca.* + 0.35 V. Successive increases in the concentration of DA corresponded to an increase in the peak current as well as a slight anodic shift in the peak potential until at 200 μM they were observed to be 5.17 μA and + 0.388 V respectively. The explanation for this anodic shift can be found within main manuscript.

Taking the 10% GO-SPE as an a representative example of all the GO-SPEs, Figure S3 shows that the oxidation peak current at a 20 μM UA concentration was 8.22 μA, which incrementally increased to 13.70 μA by 200 μM. There was a corresponding anodic shift in the onset potential from + 0.353 to + 0.406 V. It is clearly observable from Figure S3 that as with the 10% GO-SPE all the GO-SPEs display a greater anodic peak current than the bare SPE. This can be associated with the oxygenated species present on GO benefitting the oxygenated electro-catalytic reactions. This is further supported by the observation that the greater percentage incorporation of GO into the GO-SPE the larger the observed anodic peak current. However, as the percentage of GO within the electrode increases from 0 to 10% the activation potential for UA oxidation increases.

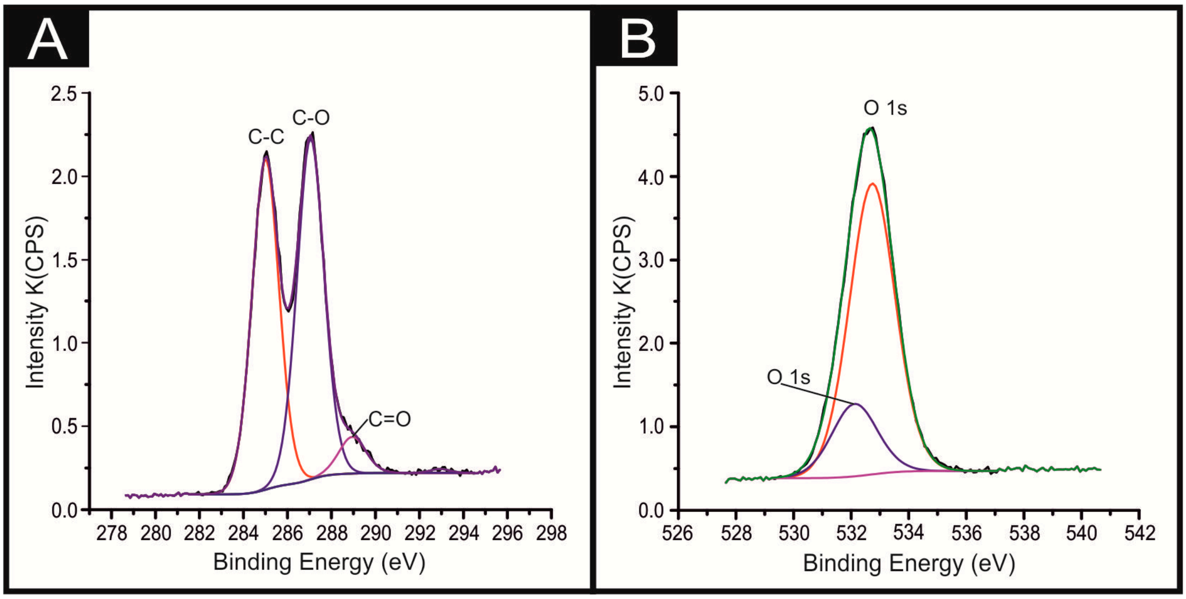

Figure S1. High-resolution XPS spectra of C and O regions of the GO utilised herein (A and B respectively).

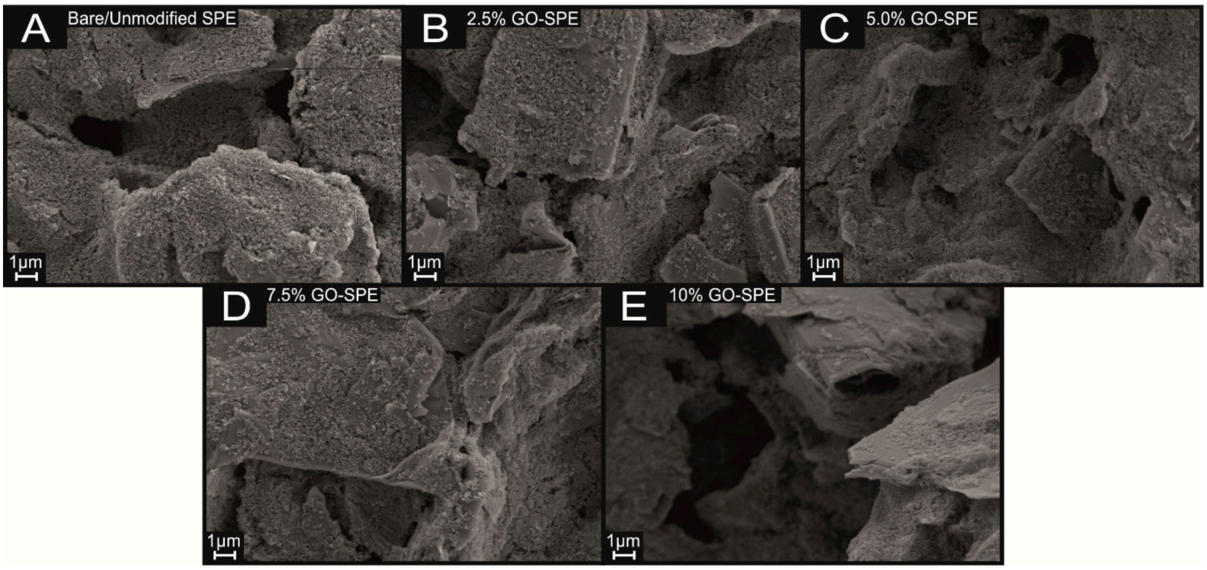

Figure S2. SEM images of the graphite and GO electrode surfaces in the supercapacitor device show little variation in the surface morphology of the surfaces with variation in GO content. Given this, it is apparent that the dominating influence of the morphology of the electrodes is in fact the carbon ink. This indicates that the improvement in the performance is a result of physicochemical properties of the graphene oxide, and not a result of any morphological differences induced by the addition of the GO.

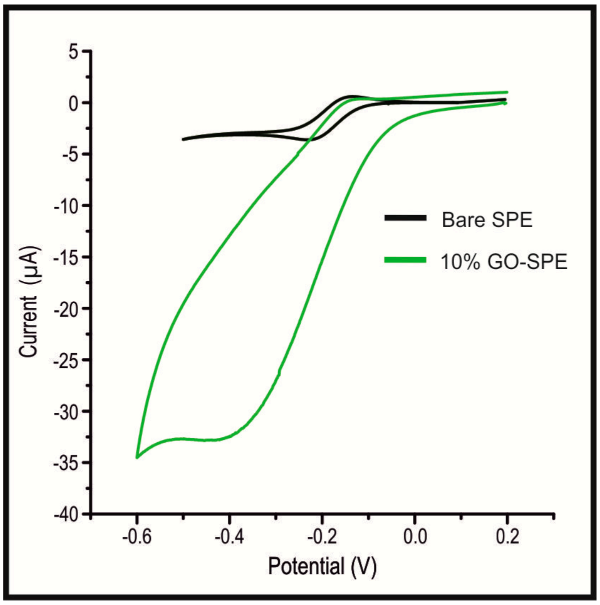

Figure S3. Typical cyclic voltammetric response of a bare SPE and a 10 % GO-SPE recorded 1 mM $[Ru(NH_3)_6]^{3+/2+}$ in 0.1 M KCl solution. Scan rate utilised: 5 mVs^{-1} (*vs.* SCE).

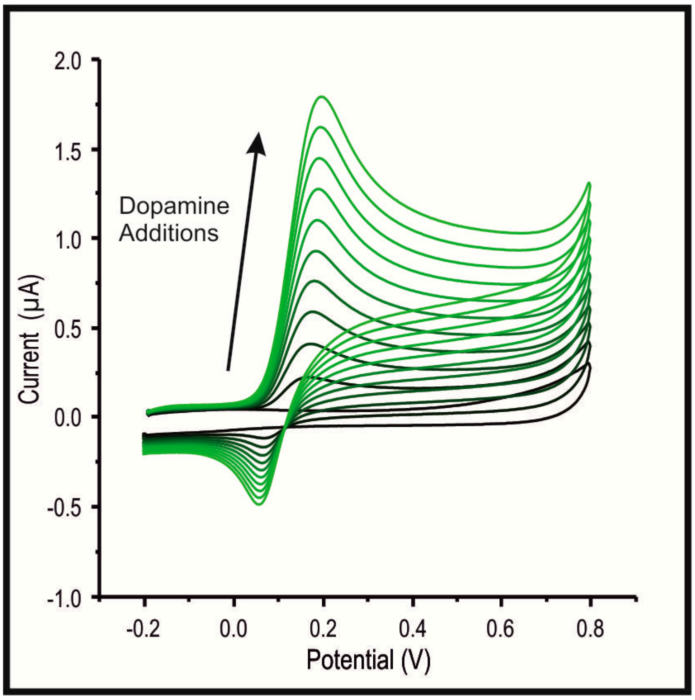

Figure S4. Typical cyclic voltammetric response obtained utilising a Bare/unmodified SPE by sequentially adding aliquots of 0.5 mM DA to pH 7.4 PBS, additions from 5 μM to 50 μM.

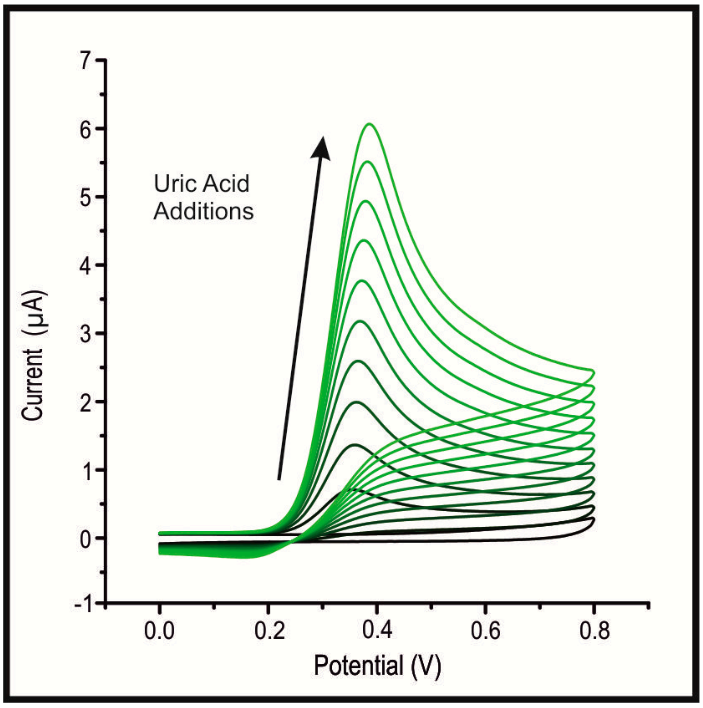

Figure S5. Typical cyclic voltammetric response obtained utilising a Bare/unmodified SPE by sequentially adding aliquots of 2 mM UA to pH 7.4 PBS, altering the bulk solution from 20 μM to 200 μM.

References

1 Choudry, N. A.; Kampouris, D. K.; Kadara, R. O.; Banks, C. E. Disposable highly ordered pyrolytic graphite-like electrodes: Tailoring the electrochemical reactivity of screen printed electrodes. *Electrochem. Commun.* **2010,** *12*, 6–9.

2 Galdino, F. E.; Foster, C. W.; Bonacin, J. A.; Banks, C. E. Exploring the electrical wiring of screen-printed configurations utilised in electroanalysis. *Anal. Methods* **2015,** *7*, 1208–1214.

3 Rowley-Neale, S. J.; Brownson, D. A. C.; Banks, C. E. Defining the origins of electron transfer at screen-printed graphene-like and graphite electrodes: MoO2 nanowire fabrication on edge plane sites reveals electrochemical insights. *Nanoscale* **2016,** *8*, 15241–15251, doi: 10.1039/C6NR04220A.

4 Yang, C.-C. Synthesis and characterization of active materials of Ni(OH)2 powders. *Int. J. Hydrogen Energy* **2002,** *27*, 1071–1081, doi: 10.1016/S0360-3199(02)00013-7.

5 Seah, M. P.; Spencer, S. J. Repeatable intensity calibration of an X-ray photoelectron spectrometer. *J. Electron. Spectrosc. Relat. Phenom.* **2006,** *151*, 178–181, doi: http://dx.doi.org/10.1016/j.elspec.2005.12.004.

6 Scofield, J. H. Hartree-Slater subshell photoionization cross-sections at 1254 and 1487 eV. *J. Electron. Spectrosc. Relat. Phenom.* **1976,** *8*, 129–137, doi: http://dx.doi.org/10.1016/0368-2048(76)80015-1.

7 Bard, A. J.; Faulkner, L. R. *Electrochemical Methods: Fundamentals and Applications*, 2 ed.; Wiley: Hoboken, NJ, USA, 2001.

© 2020 by the authors. Licensee MDPI, Basel, Switzerland. This article is an open access article distributed under the terms and conditions of the Creative Commons Attribution (CC BY) license (http://creativecommons.org/licenses/by/4.0/).
